# Iron accumulation in the ventral tegmental area in Parkinson's disease

**DOI:** 10.3389/fnagi.2023.1187684

**Published:** 2023-06-28

**Authors:** Dongling Zhang, Junye Yao, Junyan Sun, Junling Wang, Lili Chen, Hongjian He, Tao Wu

**Affiliations:** ^1^Center for Movement Disorders, Department of Neurology, Beijing Tiantan Hospital, Capital Medical University, Beijing, China; ^2^China National Clinical Research Center for Neurological Diseases, Beijing, China; ^3^Parkinson's Disease Center, Beijing Institute for Brain Disorders, Capital Medical University, Beijing, China; ^4^Center for Brain Imaging Science and Technology, College of Biomedical Engineering and Instrument Science, Zhejiang University, Hangzhou, Zhejiang, China; ^5^School of Physics, Zhejiang University, Hangzhou, Zhejiang, China

**Keywords:** Parkinson's disease, ventral tegmental area (VTA), idiopathic rapid eye movement sleep behavior disorder (RBD), iron deposition, quantitative susceptibility mapping

## Abstract

**Introduction:**

The ventral tegmental area (VTA) is less affected compared to substantia nigra pars compacta (SNc) in Parkinson's disease (PD). This study aimed to quantitatively evaluate iron content in the VTA across different stages of PD in order to help explain the selective loss of dopamine neurons in PD.

**Methods:**

Quantitative susceptibility mapping (QSM) data were obtained from 101 PD patients, 35 idiopathic rapid eye movement sleep behavior disorder (RBD) patients, and 62 healthy controls (HCs). The mean QSM values in the VTA and SNc were calculated and compared among the groups.

**Results:**

Both RBD and PD patients had increased iron values in the bilateral SNc compared with HCs. RBD and PD patients in the Hoehn–Yahr (H & Y) stage 1 did not show elevated iron values in the VTA, while PD patients with more than 1.5 H & Y staging had increased iron values in bilateral VTA compared to HCs.

**Discussion:**

This study shows that there is no increased iron accumulation in the VTA during the prodromal and early clinical stages of PD, but iron deposition increases significantly as the disease becomes more severe.

## 1. Introduction

The dopamine (DA) neurons in the ventral tegmental area (VTA) principally project to the nucleus accumbens in the ventral striatum, as well as the amygdala and prefrontal cortex, as part of the mesocorticolimbic pathway, whereas the DA neurons in the substantia nigra pars compacta (SNc) mainly project to the dorsal striatum, as part of the nigrostriatal pathway. The mesocorticolimbic pathway involves a variety of behaviors and psychopathological states, such as depression, anxiety, feeding, and reward-related and goal-directed behaviors (Alberico et al., 2015). In Parkinson's disease (PD), an impaired dopaminergic mesocorticolimbic system is considered the leading cause of neuropsychiatric symptoms (Castrioto et al., 2016). In PD, degeneration of the DA neurons in the SNc is the most prominent symptom; in contrast, the DA neurons in the VTA are less affected (Ropper et al., 2019). Although some theories have been suggested, such as the variety of neurons found in the VTA (Nair-Roberts et al., 2008), lower expression of the dopamine transporters (Lammel et al., 2008), differences in calcium channel expression (Mosharov et al., 2009), levels of cytosolic DA, and the presence of α-synuclein (Mosharov et al., 2009; Pan and Ryan, 2012), it remains unclear why the VTA is relatively spared in PD.

It has been demonstrated that abnormal iron deposition may contribute to the damage of DA neurons in PD (Hare and Double, 2016). Quantitative susceptibility mapping (QSM) is more sensitive and can better detect increased iron in PD than R2 and R2^*^ mapping (Barbosa et al., 2015). Using QSM, several research studies have shown that iron accumulation was both cross-sectionally and longitudinally increased in the substantia nigra (SN) in PD patients, and iron levels were correlated with clinical manifestations, using QSM (Bergsland et al., 2019; Sun et al., 2020; Uchida et al., 2020). In contrast, our knowledge of iron accumulation in the VTA in PD remains limited. A study on chronic 1-methyl-4-phenyl-1,2,3,6-tetrahydropyridine (MPTP)-treated PD mice detected an increased iron level in the SNc, but not in the VTA, and suggested that difference in iron deposition might be a reason contributing to the selective degeneration of DA neurons (Lv et al., 2011). So far, only one imaging study has investigated iron accumulation in the VTA in PD patients and found increased iron content (Ahmadi et al., 2020). However, as the primary purpose was to use transcranial sonography and QSM to localize the SN, the previous study did not investigate the iron levels at prodromal and different clinical stages of PD or the relationship between iron levels and clinical characteristics.

Thus, it is still unclear if increased iron deposition occurs in the VTA during the prodromal and early clinical stage of PD, whether iron accumulation increases as the disease becomes more severe, and whether iron deposition in the VTA correlates with clinical manifestations. Therefore, this study aimed to quantitatively evaluate iron contents in the VTA across the prodromal and different clinical stages of PD. Idiopathic rapid eye movement sleep behavior disorder (RBD) patients were included in the current study to evaluate iron content in the prodromal stage. RBD is considered a prodromal stage of α-synucleinopathies since RBD patients have a high rate of conversion to neurodegenerative disorders, especially α-synucleinopathies, such as PD, dementia with Lewy bodies, and multiple system atrophy (Schenck et al., 2013). We hypothesized that iron content in the VTA is not elevated in the early stage of PD but gradually increased as the disease becomes more severe. This study will help to clarify the pattern of iron deposition in the VTA and may provide an explanation of the selective damage of DA neurons in PD.

## 2. Materials and methods

### 2.1. Participants

This experiment was performed in accordance with the Declaration of Helsinki and was approved by the Institutional Review Board of Xuanwu Hospital of Capital Medical University. All participants (35 RBD patients, 101 PD patients, and 62 HCs) provided written consent before the experiment and were recruited from the Movement Disorders Clinic of the Xuanwu Hospital of Capital Medical University. The RBD patients were screened by the International Classification of Sleep Disorder-Third Edition diagnostic criteria (American Academy of Sleep Medicine, 2014) and were confirmed by polysomnography. PD patients were diagnosed by the MDS Clinical Diagnostic Criteria (Andrew et al., 1992). The inclusion criteria for HCs were (1) no family history of movement disorders, (2) no neurological or psychiatric diseases, and (3) no obvious cerebral lesions on structural images in magnetic resonance imaging (MRI).

The PD patients were evaluated using the Movement Disorder Society (MDS) Unified Parkinson's Disease Rating Scale, Part III (MDS-UPDRS III) and Hoehn and Yahr (H & Y) stage while off their anti-parkinsonian medicine for 12 h. The RBD patients were assessed by the Rapid Eye Movement Sleep Behavior Disorder Questionnaire–Hong Kong (RBDQ-HK) and the MDS-UPDRS III. In addition, all participants were evaluated using the Hamilton Depression Scale (HAMD), Montreal Cognitive Assessment (Chinese version; C-MoCA), Non-Motor Symptoms Scale for Parkinson's Disease (NMSS), Brief Smell Identification Test (BSIT), Epworth Sleepiness Scale (ESS), Pittsburgh Sleep Quality Index (PSQI), and Apathy Scale (AS). Demographic information is summarized in Table 1.

**Table 1:** Demographic and clinical data of participants and the QSM values in ROIs.

	**HC tblhead(mean ± SD) tblhead*n* = 62**	**RBD tblhead(mean ± SD) tblhead*n* = 35**	**PD (mean ±SD) tblhead*n* = 101**	***P***	***P*** **(*****post-hoc*****)**		
					**HC vs. RBD**	**RBD vs. PD**	**HC vs. PD**
Age	64.97 ± 5.76	65.86 ± 6.31	64.21 ± 6.67	0.388^a^	1	0.550	1
Sex (M/F)	25/37	21/14	54/47	0.123^a^	0.062	0.503	0.103
RBDQ-HK	7.11 ± 5.75	34.44 ± 13.69	20.89 ± 16.36	**< 0.001** ^a^	**< 0.001**	**< 0.001**	**< 0.001**
HAMD	3.20 ± 3.10	5.76 ± 4.04	5.67 ± 3.96	**< 0.001** ^a^	**0.024**	1	**< 0.001**
C-MoCA	26.00 ± 2.28	25.11 ± 2.84	23.76 ± 3.74	**0.001** ^a^	0.781	0.212	**< 0.001**
NMSS	14.04 ± 13.21	21.65 ± 13.54	38.77 ± 33.79	**< 0.001** ^a^	0.688	**0.013**	**< 0.001**
BSIT	8.86 ± 2.48	7.37 ± 2.13	7.27 ± 2.91	**0.002** ^a^	0.052	1	**0.002**
ESS	4.26 ± 2.98	5.47 ± 3.03	5.72 ± 3.26	0.061^a^	0.394	1	0.061
PSQI	6.21 ± 5.26	7.09 ± 4.09	6.09 ± 3.70	0.518^a^	1	0.793	1
AS	6.49 ± 6.30	9.35 ± 7.57	12.48 ± 9.04	**< 0.001** ^a^	0.508	0.360	**< 0.001**
UPDRS III	-	5.09 ± 3.83	32.27 ± 13.68	-	-	-	-
Duration (year)	-	3.01 ± 1.48	4.85 ± 2.68	-	-	-	-
H & Y stage	-	-	1.97 ± 0.69	-	-	-	-
VTA_L (ppm)	0.0193 ± 0.003	0.0204 ± 0.003	0.0213 ± 0.002	**< 0.001** ^**b**^	0.174	0.341	**< 0.001**
VTA_R	0.0197 ± 0.003	0.0208 ± 0.002	0.0214 ± 0.002	**< 0.001** ^**b**^	0.137	0.570	**< 0.001**
SNc_L	0.0280 ± 0.011	0.0393 ± 0.014	0.0508 ± 0.022	**< 0.001** ^**b**^	**0.009**	**0.004**	**< 0.001**
SNc_R	0.0263 ± 0.014	0.0398 ± 0.014	0.0467 ± 0.026	**0.001** ^**b**^	**0.008**	0.240	**< 0.001**

HC, healthy control; RBD, rapid eye movement sleep behavior disorder; PD, Parkinson's disease; M, male; F, female; RBDQ-HK, Rapid Eye Movement Sleep Behavior Disorder Questionnaire–Hong Kong; HAMD, Hamilton Depression Scale; C-MoCA, Montreal Cognitive Assessment (Chinese version); NMSS, Non-Motor Symptoms Scale for Parkinson's Disease; BSIT, Brief Smell Identification Test; ESS, Epworth Sleepiness Scale; PSQI, Pittsburgh Sleep Quality Index; AS, Apathy Scale; MDS-UPDRS III, Movement Disorder Society Unified Parkinson's Disease Rating Scale, Part III; VTA, ventral tegmental area; SNc, substantia nigra pars compacta; ppm, parts per million; L, left; R, right.

^a^ANOVA, analysis of variance.

^b^ANCOVA, analysis of covariance.

-, Not applicable. Bold values: *P* < 0.05.

### 2.2. MRI data collection

MRI data were collected on a 3T MAGNETOM Skyra scanner (Siemens, Erlangen, Germany) using a 20-channel head coil. The signals from different coils were combined by the sum of squares method. A single-echo 3-dimensional (3-D) gradient echo (GRE) sequence was collected with the following parameters: voxel size = 0.667 × 0.667 × 1.5 mm3, repetition time (TR) = 25 ms, echo time (TE) = 17.5 ms, slice thickness = 1.5 mm, flip angle = 15°, field of view (FoV) = 256 × 192 mm^2^, and scanning time = 5 min 6 s. A whole-brain sagittal 3-D T1 magnetization-prepared rapid gradient echo (MP-RAGE) imaging was performed with the following parameter: voxel size = 1 × 1 × 1 mm3, TR = 2,530 ms, TE = 2.98 ms, TI = 1,100 ms; slice thickness = 1 mm, flip angle = 7°, FoV = 256 × 224 mm^2^, and scanning time = 5 min 13 s.

### 2.3. Image analysis

The QSM reconstruction was performed using MATLAB 2017b based STI Suite.^1^ The phase images were unwrapped using a Laplacian-based algorithm method (Wu et al., 2012). The unwrapped phase images were used to remove the background field using the V-SHARP method (Li et al., 2014). The magnetic susceptibility was determined using streaking artifact reduction for QSM (STAR-QSM; Wei et al., 2015).

Image registration was performed using FMRIB Software Library (FSL) v6.0.^2^ Individual 3D-T1 images were first skull stripped and registered to a standard space [Montreal Neurological Institute (MNI) 152] using FSL's FLIRT and FNIRT tools. The inverted warping field from standard to native space was then obtained by inverting the warping field. Thereafter, the individual 3D-T1 image was also registered to GRE's magnitude image using the FLIRT tool to get a second warping field. Both warping fields were combined to converted to obtain warping fields covert MNI152 so that it was well-coregistered with individual's susceptibility map.

**Figure 1:**
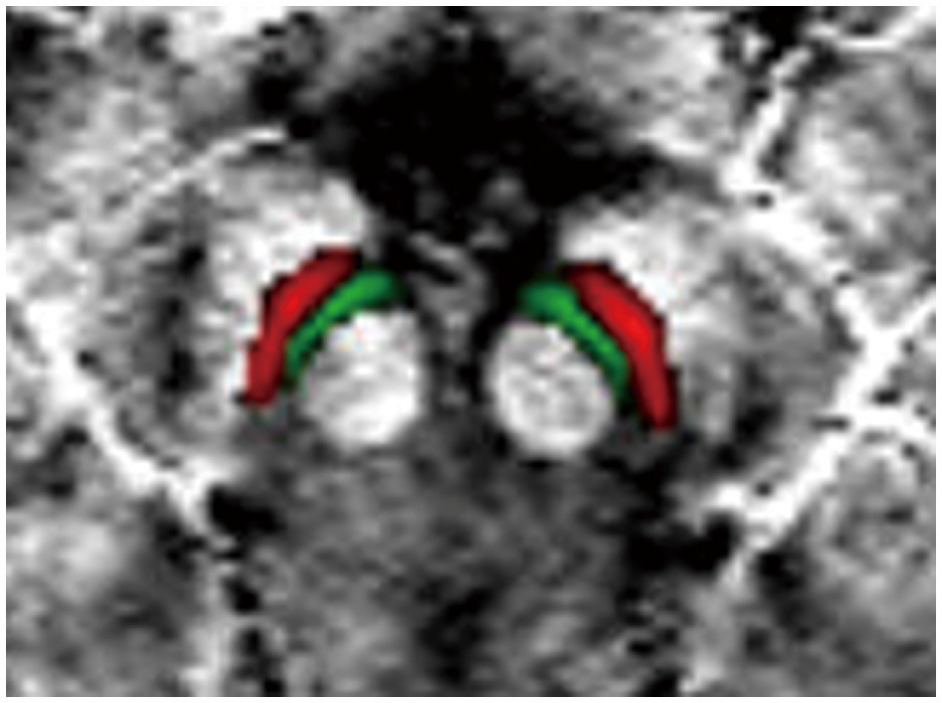
Definition of regions of interest (ROIs). The ROIs include the VTA (green) and SNc (red). VTA, ventral tegmental area; SNc, substantia nigra pars compacta.

The VTA and SNc were defined by the California Institute of Technology (CIT) 168 atlas of subcortical nuclei (Pauli et al., 2018), with a threshold of 0.25. The CIT168 atlas divides the VTA into the parabrachial pigmented nucleus (PBP) and VTA nucleus. Using FSL, we merged the VTA nucleus and PBP into a whole VTA for two reasons: (1) there is little evidence that the VTA component nuclei represent neural populations specialized and distinct in function (Trutti et al., 2019) and (2) as a probabilistic atlas (Pauli et al., 2018), there is some overlap between the VTA nucleus and PBP. The bilateral SNc and merged VTA were used as the ROIs in the current study (Figure 1). The ROIs in standard MNI152 space were normalized to individual magnitude space using the above-mentioned warping fields using FSL. Finally, the individual ROIs were obtained in order to calculate QSM values.

### 2.4. Statistical analyses

The demographic and clinical characteristics of HC, RBD, and PD groups were compared using the analysis of variance (ANOVA). *Post-hoc* tests with Bonferroni correction were used for intergroup comparisons. The Pearson chi-square test was applied for sex frequency among the groups.

The normal distribution of QSM values was confirmed using the one-sample Kolmogorov–Smirnov test. The differences in QSM values among the three groups in each ROI were analyzed using the analysis of covariance (ANCOVA) with age and sex as covariables. *Post-hoc* tests with Bonferroni correction were used for intergroup comparisons (*P* < 0.05). In order to reveal the iron levels in different stages of PD, we further divided our PD patients into three subgroups according to the H & Y stage: 26 PD patients with H & Y stage 1 (PD-H&Y1), 43 PD patients with H & Y stage 1.5 and 2 (PD-H&Y2, including four patients with H & Y stage 1.5), and 32 PD patients with H & Y stage 2.5 and 3 (PD-H&Y3, including 14 patients with H & Y stage 2.5). The differences in QSM values among the HCs and three PD subgroups in each ROI were also analyzed using ANCOVA. *Post-hoc* tests with Bonferroni correction were used for intergroup comparisons (*P* < 0.05).

In addition, we calculated the mean QSM values of the bilateral VTA, as well as the differences in mean QSM values between the groups and the different H & Y stages.

Correlations between QSM values and clinical assessments in RBD and PD patients were performed using Pearson's correlation analysis, while Spearman's correlation analysis was used to analyze the correlation between H & Y stage and QSM values in PD patients. Statistical analyses were performed using IBM SPSS Statistics (version 20, IBM Corp, Armonk, NY, USA).

## 3. Results

No significant differences were observed among the three groups in age, sex, and PSQI (ANOVA, *P* > 0.05), while there were significant differences in RBDQ-HK, HAMD, C-MoCA, NMSS, BSIT, ESS, and AS scores (ANOVA, *P* < 0.05; Table 1).

**Figure 2:**
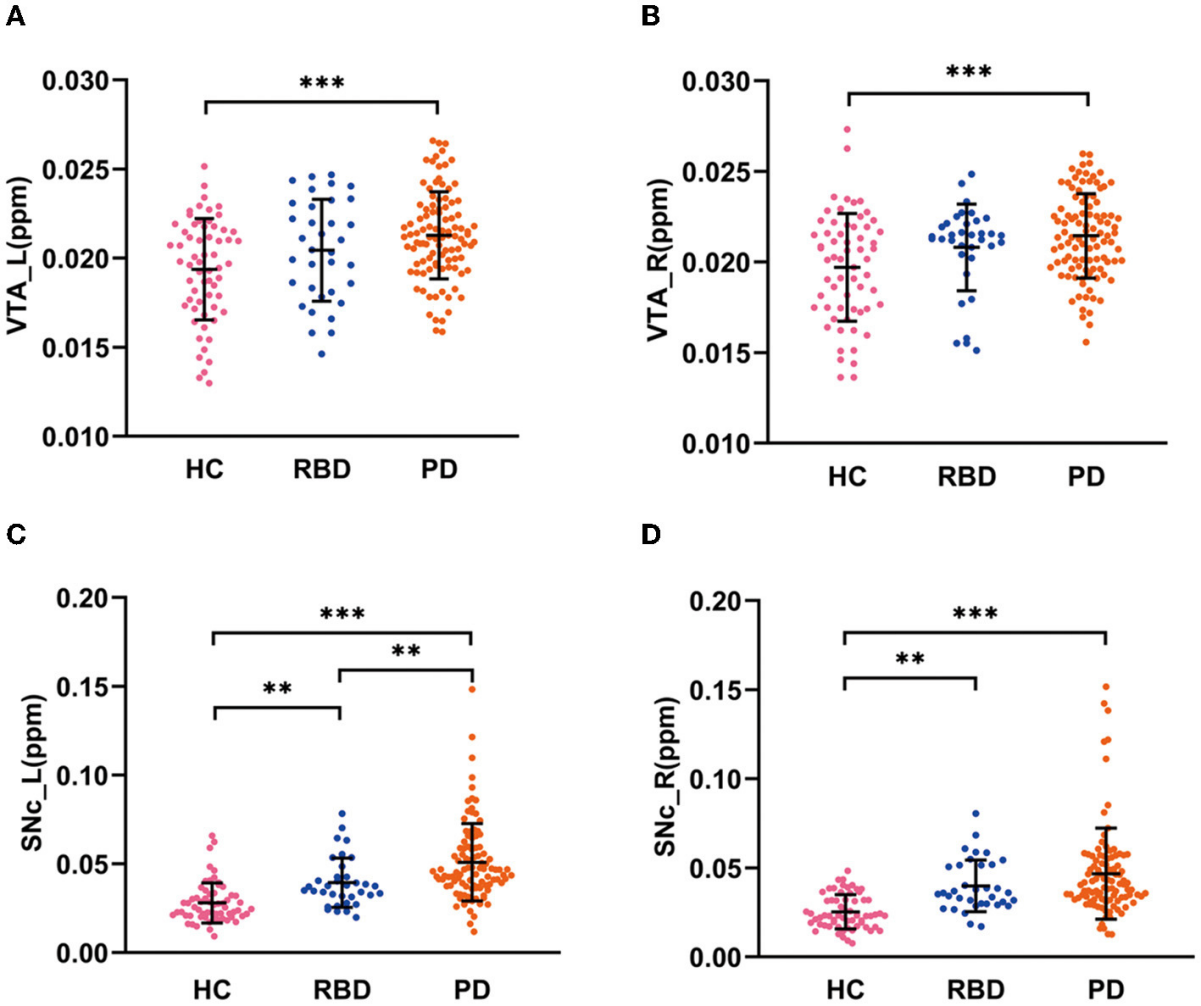
QSM values in the VTA and SNc in three groups. **(A, B)** QSM values in the VTA in HC, RBD, and PD groups. **(C, D)** QSM values in the SNc in HC, RBD, and PD groups. HC, healthy control; RBD, idiopathic rapid eye movement sleep behavior disorder; PD, Parkinson's disease. VTA, ventral tegmental area; SNc, substantia nigra pars compacta; ppm, parts per million; L, left; R, right. ***P < 0.001; **P < 0.01.

There were significant differences in QSM values in the bilateral VTA and SNc among the HC, RBD, and PD groups (ANCOVA, *P* < 0.001). The RBD group did not show enhanced iron values in the bilateral VTA (*post-hoc* test, *P* > 0.05, Bonferroni corrected), but had increased iron values in the bilateral SNc (*post-hoc* test, *P* < 0.01, Bonferroni corrected) compared with HCs. PD patients had increased iron values in the bilateral VTA and SNc compared with HCs (*post-hoc* test, *P* < 0.001, Bonferroni corrected) and had enhanced iron values in the left SNc compared with RBD patients (*post-hoc* test, *P* < 0.01, Bonferroni corrected; Table 1 and Figures 2A–D).

**Figure 3:**
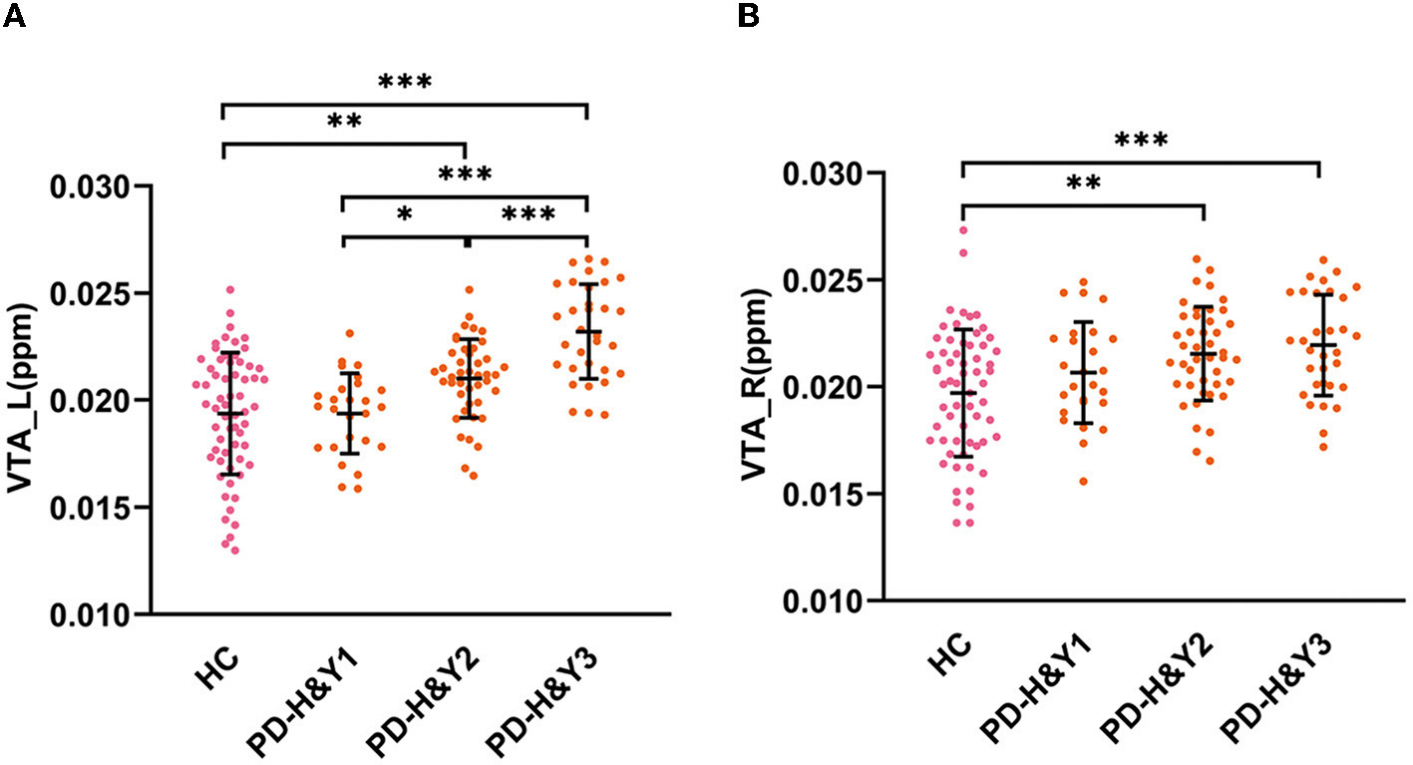
QSM values in the VTA in HCs and PD patients with different H & Y stages. **(A)** The QSM values in the left VTA; **(B)** the QSM values in the right VTA; HC, healthy control; PD, Parkinson's disease; H & Y stage, Hoehn and Yahr stage; VTA, ventral tegmental area; ppm, parts per million; L, left; R, right. ***P < 0.001; **P < 0.01; *P < 0.05.

The QSM values had significant differences among the HCs and three PD subgroups in the bilateral VTA and SNc (ANCOVA, *P* < 0.001). In the bilateral VTA, the PD-H&Y1 group did not show significantly higher iron values than the HC group (*post-hoc* test, *P* > 0.05, Bonferroni corrected), while the PD-H&Y2 and PD-H&Y3 groups showed higher values (*post-hoc* test, *P* < 0.01, Bonferroni corrected). In the left VTA, the PD-H&Y3 group had increased iron values compared with the PD-H&Y1 and PD-H&Y2 groups (*post-hoc* test, *P* < 0.001, Bonferroni corrected), while the PD-H&Y2 group also had an increase of iron values compared with the PD-H&Y1 group (*post-hoc* test, *P* < 0.05, Bonferroni corrected). In comparison to HCs, all three PD subgroups showed higher iron values in the bilateral SNc (*post-hoc* test, *P* < 0.05, Bonferroni corrected). The PD-H&Y3 group had increased iron values in the left SNc compared with the PD-H&Y1 and PD-H&Y2 groups (*post-hoc* test, *P* < 0.001, Bonferroni corrected) and had elevated iron values in the right SNc compared with the PD-H&Y1 group (*post-hoc* test, *P* < 0.05, Bonferroni corrected; Table 2 and Figures 3A, B).

**Table 2:** QSM values of HC and PD patients with different H & Y stages in ROIs.

	**HC**	**PD-H&Y1**	**PD-H&Y2**	**PD-H&Y3**	**ANCOVA**	***P*** **(*****post-hoc*****)**					
	**(mean** ±**SD)**	**(mean** ±**SD)**	**(mean** ±**SD)**	**(mean** ±**SD)**	***P***	**HC vs. PD-H&Y1**	**HC vs. PD-H&Y2**	**HC vs. PD-H&Y3**	**PD-H&Y1 vs. H&Y2**	**PD-H&Y2 vs. H&Y3**	**PD-H&Y1 vs. H&Y3**
VTA_L	0.0193 ± 0.003	0.0194 ± 0.002	0.0210 ± 0.002	0.0232 ± 0.002	**< 0.001**	1	**0.004**	**< 0.001**	**0.037**	**0.001**	**< 0.001**
VTA_R	0.0197 ± 0.003	0.0207 ± 0.002	0.0215 ± 0.002	0.0219 ± 0.002	**< 0.001**	1	**0.004**	**0.001**	0.750	1	0.284
SNc_L	0.0280 ± 0.011	0.0414 ± 0.015	0.0450 ± 0.016	0.0661 ± 0.026	**< 0.001**	**0.011**	**< 0.001**	**< 0.001**	1	**< 0.001**	**< 0.001**
SNc_R	0.0253 ± 0.010	0.0394 ± 0.013	0.0441 ± 0.023	0.0554 ± 0.030	**< 0.001**	**0.035**	**< 0.001**	**< 0.001**	1	0.126	**0.019**

HC, healthy control; PD, Parkinson's disease; VTA, ventral tegmental area; SNc, substantia nigra pars compacta; L, left; R, right; ANCOVA, analysis of covariance. Bold values: *P* < 0.05.

We found significant differences in mean VTA QSM values between the groups and at different stages, which was similar to the results of the left and right VTA QSM values (Supplementary Tables 1, 2).

**Figure 4:**
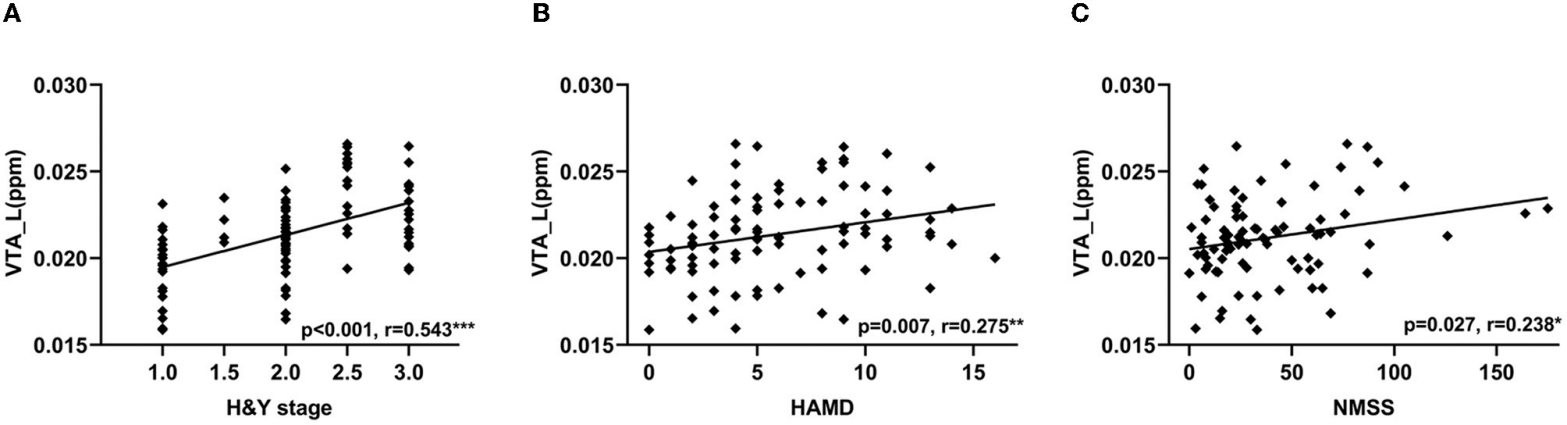
Correlations between QSM values in the VTA and clinical features in PD patients. Correlation between QSM values in the left VTA and H & Y stage **(A)**, HAMD **(B)**, and NMSS scores **(C)**. VTA, ventral tegmental area; QSM, quantitative susceptibility mapping; PD, Parkinson's disease; H & Y stage, Hoehn and Yahr stage; HAMD, Hamilton Depression Scale; NMSS, Non-Motor Symptoms Scale for Parkinson's Disease. ***P < 0.001; **P < 0.01; *P < 0.05.

In PD patients, the QSM values in the left VTA were positively correlated with H & Y stage (*r* = 0.543, *p* < 0.001, Figure 4A), HAMD scores (*r* = 0.275, *p* = 0.007, Figure 4B), and NMSS (*r* = 0.238, *p* = 0.027, Figure 4C). In RBD patients, QSM values in the left SNc were positively correlated with disease duration (*r* = 0.356, *p* = 0.045) and RBDQ-HK scores (*r* = 0.388, *p* = 0.023), while QSM values in the right SNc were negatively correlated with BSIT scores (*r* = −0.496, *p* = 0.008). The QSM values in the bilateral SNc were positively correlated with disease duration (left: *r* = 0.306, *p* = 0.002; right: *r* = 0.211, *p* = 0.034). In addition, the QSM values in the left SNc were positively correlated with the H & Y stage (*r* = 0.462, *p* < 0.001), MDS-UPDRS III (*r* = 0.250, *p* = 0.013), and HAMD scores (*r* = 0.233, *p* = 0.022), while the QSM values in the right SNc were positively correlated with the AS scores (*r* = 0.226, *p* = 0.045) in PD patients.

## 4. Discussion

In the current study, we investigated iron accumulation in the VTA across different stages of PD. The novel finding is that iron contents are not increased in the RBD patients and PD patients at H & Y stage 1. The iron accumulation in the VTA becomes significant in PD patients at mid stages and advanced stages. QSM values in the left VTA positively correlate with the H & Y stage, NMSS, and HAMD scores in PD patients.

We found that both RBD and PD patients had enhanced iron contents in the bilateral SNc, which is consistent with previous reports (Guan et al., 2017; Sun et al., 2020). In contrast, there was no increased iron content in the VTA in RBD patients and PD patients at H & Y stage 1. Age-related iron accumulation might be an important factor contributing to neurodegeneration, as aging processes might compromise the iron homoeostatic system, leading to an excess of iron that is not efficiently chelated by storage proteins or other molecules (Killilea et al., 2004; Ward et al., 2014). An elevated level of iron deposition in PD may result from increased iron influx (Moos et al., 2007), loss of intracellular homeostasis (Zucca et al., 2017), or impaired iron efflux (Bonaccorsi di Patti et al., 2018). The interaction between excess iron and DA can produce neurotoxic intermediate or end-products, leading to the formation of DNA adducts, lipid peroxidation, loss of membrane integrity, and induction of apoptosis (Blum et al., 2001; Hare and Double, 2016). It has been approved that α-synuclein could form toxic aggregates in the presence of iron, which is considered to contribute to the formation of Lewy bodies in DA neurons via oxidative stress (Ostrerova-Golts et al., 2000). It has been proposed that the vulnerability of DA neurons requires redox load from a combination of relatively high iron and dopamine together (Hare et al., 2014). Therefore, increased iron deposition is believed to mediate the death of SNc dopaminergic neurons (Hare and Double, 2016). Previous studies have suggested that several reasons may relate to less damaged DA neurons in the VTA compared with SNc, such as the variety of neurons found in the VTA (Nair-Roberts et al., 2008), lower expression of the dopamine transporter (Lammel et al., 2008), differences in calcium channel expression and the presence of α-synuclein (Mosharov et al., 2009), differences in vesicular monoamine transporter-2 and neuromelanin (Liang et al., 2004), less degree of oxidative stress and more inducible copper-zinc superoxide dismutase activities (Hung and Lee, 1998), and more brain-derived neurotrophic factor mRNA gene expression (Hung and Lee, 1996). According to our findings, less accumulation of iron is also a likely reason contributing to the relatively spared DA neurons in the VTA during the prodromal and early clinical stages of PD.

The underlying reasons contributing to the less accumulation of iron in the VTA compared with SNc remain unclear. Previous studies on chronic MPTP-treated mice have suggested that misregulation of iron transporters, such as increased expression of divalent metal transporter 1 and decreased expression of ferroportin 1, might correlate with nigral iron accumulation. However, this pattern of misregulation of iron transporters was not detected in the VTA (Lv et al., 2011). These selective changes in iron transporters may help explain the differential iron accumulation in the SNc and VTA in PD.

Our PD patients at more than 1.5 H & Y staging had enhanced iron deposition in the VTA. This finding is consistent with a previous report (Ahmadi et al., 2020), in which PD patients with an average of 1.97 H & Y staging showed increased iron accumulation in the VTA compared with HC. In addition, the QSM values in the left VTA were positively correlated with the H & Y stage. These observations suggest that as the disease becomes more severe, iron deposition in the VTA becomes more significant, and may induce the death of DA neurons at the mid-stage and advanced stage of PD. Studies on the post-mortem brain of PD patients have proved that there was a 40–77% loss of DA neurons in the VTA (Alberico et al., 2015).

The neurons in the VTA project to extensive brain regions, including the nucleus accumbens, amygdala, prefrontal cortex, hippocampus, ventral pallidum, periaqueductal gray, bed nucleus of the stria terminalis, olfactory tubercle, and locus coeruleus, which are related to the various non-motor symptoms (Alberico et al., 2015; Morales and Margolis, 2017). However, only a small number of imaging studies have focused on the relationship between physiological changes in the VTA and clinical phenotypes of PD. The VTA showed an attenuated neural response to reward outcomes in PD patients (van der Vegt et al., 2013). Increased functional coupling between the VTA and default mode network has been reported in PD patients with freezing of gait (Steidel et al., 2021). In addition, increased functional connectivity between the VTA and anterior cingulate cortex was related to depression in PD (Wei et al., 2018). We found that the QSM values in the left VTA were positively correlated with NMSS and HAMD scores, which provides further support that damaged VTA is a reason contributing to non-motor symptoms, especially depression, in PD patients.

We found that only the iron contents of left VTA, not right VTA, were significantly correlated with clinical symptoms in PD patients. This phenomenon is likely due to the asymmetry of motor symptoms as most of our PD patients had right-side onset (65 of 101 patients). As our RBD patients did not show significant motor symptoms, we could not define the more- and less-affected sides in RBD patients. Thus, we only performed between-group comparisons of QSM values on the left and right sides.

In RBD patients, the QSM values in the SNc were positively correlated with disease duration, which is consistent with our previous report (Sun et al., 2020) and indicates that iron deposition in the SNc increases with the progression of RBD. In addition, the QSM values in the SNc correlated with RBDQ-HK and BSIT scores. Olfactory dysfunction is associated with an increased risk of developing PD, and RBD patients with hyposmia are at high risk for converting to PD (Lyu et al., 2021). These results suggest that iron accumulation in the SNc is associated with the severity of RBD and may have the potential to predict the conversion to α-synucleinopathies, which needs to be proved in future longitudinal studies.

In the bilateral SNc, PD patients had significantly enhanced iron contents, which is consistent with previous studies (He et al., 2015; Guan et al., 2017; Bergsland et al., 2019; Ahmadi et al., 2020; Sun et al., 2020; Fu et al., 2021), and the QSM values were positively correlated with disease duration and the H & Y stage (Du et al., 2016; Fu et al., 2021). The QSM values were positively correlated with the MDS-UPDRS III and HAMD scores in the left SNc (He et al., 2015; Fu et al., 2021) as well as the AS scores in the right SNc. The enhanced iron might aggravate the dysfunction of the nigrostriatal pathway with disease progression and severity (Hare and Double, 2016), which exacerbates the motor and non-motor symptoms. These findings suggest that the QSM technique has the potential to be a neuroimaging marker of disease progression, which needs to be examined in future longitudinal studies.

There are some limitations in our study. First, this is a cross-sectional study, and longitudinal studies are needed to reveal the progress of iron deposition in the VTA and its relationship with clinical progression. Second, as we only had a small number of patients at the H & Y stage 4, the iron accumulation in the VTA in more advanced PD patients was not investigated in the current study.

## 5. Conclusion

Using the QSM, we demonstrate that the iron content in the VTA is not enhanced in the prodromal and early clinical stage of PD but becomes significantly increased as the disorder becomes more severe. Moreover, the iron deposition in the VTA is associated with the non-motor symptoms in PD. Our findings may help to understand the iron deposition in the VTA at different stages of PD and its relationship with clinical manifestations of PD.

## Data availability statement

The raw data supporting the conclusions of this article will be made available by the authors, without undue reservation.

## Ethics statement

The studies involving human participants were reviewed and approved by Institutional Review Board of Xuanwu Hospital of Capital Medical University (2011-27). The patients/participants provided their written informed consent to participate in this study.

## Author contributions

DZ and JY contributed to the organization and execution of the research project. DZ drafted the manuscript. DZ, JY, JS, JW, and LC contributed to the acquisition, post-processing, and analysis of the data. TW and HH revised the manuscript. All authors contributed to the article and approved the submitted version.

## Footnotes

^1^ https://people.eecs.berkeley.edu/~chunlei.liu/software.html

^2^ https://fsl.fmrib.ox.ac.uk/fsl/fslwiki/
