# Supplementary material for: Iron accumulation in the ventral tegmental area in Parkinson's disease
Source: Front Aging Neurosci. 2023 Jun 28;15:1187684. doi: 10.3389/fnagi.2023.1187684 (PMC10338054; doi:10.3389/fnagi.2023.1187684)
Supplement: Supplementary file 1 [file Table_1.DOCX]

**Table 1. The mean QSM values in ROIs in HC, RBD and PD groups.**

|  | HC  (mean ± SD)  n=62 | RBD  (mean ± SD)  n=35 | PD  (mean ± SD)  n=101 | ancova  P | P (post hoc) | | |
| --- | --- | --- | --- | --- | --- | --- | --- |
|  |  |  |  |  | HC vs RBD | RBD vs PD | HC vs PD |
| VTA_mean | 0.0195±0.003 | 0.0206±0.002 | 0.0214±0.002 | **0.001** | 0.112 | 0.182 | **<0.001** |

HC, healthy control; RBD, rapid eye movement sleep behavior disorder; PD, Parkinson’s disease; VTA, ventral tegmental area; SNc, substantia nigra pars compacta; ppm, parts per million; L, left; R, right.

**Table 2. The mean QSM values of HC and PD patients with different H&Y stage in ROIs**

|  | HC | PD-H&Y1 | PD-H&Y2 | PD-H&Y3 | ancova  P | P (post hoc) | | | | | |
| --- | --- | --- | --- | --- | --- | --- | --- | --- | --- | --- | --- |
|  | (mean ± SD) | (mean ± SD) | (mean ± SD) | (mean ± SD) |  | HC vs  PD-H&Y1 | HC vs  PD-H&Y2 | HC vs  PD-H&Y3 | PD-H&Y1 vs H&Y2 | PD-H&Y2 vs H&Y3 | PD-H&Y1 vs H&Y3 |
|  | n=62 | n=26 | n=43 | n=32 |  |  |  |  |  |  |  |
| VTA_mean | 0.0195±0.003 | 0.0200±0.002 | 0.0213±0.001 | 0.0226±0.002 | **<0.001** | 1 | **<0.001** | **<0.001** | 0.068 | **0.038** | **<0.001** |

HC, healthy control; PD, Parkinson’s disease; VTA, ventral tegmental area; SNc, substantia nigra pars compacta; L, left; R, right.
